# Association between levels of persistent organic pollutants in adipose tissue and cryptorchidism in early childhood: a case–control study

**DOI:** 10.1186/s12940-015-0065-0

**Published:** 2015-09-24

**Authors:** Jaakko J. Koskenniemi, Helena E. Virtanen, Hannu Kiviranta, Ida N. Damgaard, Jaakko Matomäki, Jørgen M. Thorup, Timo Hurme, Niels E. Skakkebaek, Katharina M. Main, Jorma Toppari

**Affiliations:** Departments of Physiology and Paediatrics, University of Turku and Turku University Hospital, Kiinamyllynkatu 10, FI-20520 Turku, Finland; Department of Growth and Reproduction and EDMaRC, Rigshospitalet, Blegdamsvej 9, DK-2100 Copenhagen, Denmark; National Institute for Health and Welfare, Department of Health Protection, P.O. Box 95, FI-70701 Kuopio, Finland; Clinical Research Center, Turku University Hospital, Kiinamyllynkatu 4-8 PL 52, FI-20521 Turku, Finland; The Department of Paediatric Surgery, Rigshospitalet, Blegdamsvej 9, DK-2100 Copenhagen, Denmark; Faculty of Health and Medical Science, University of Copenhagen, Blegdamsvej 3B, DK-2200 Copenhagen, Denmark; Department of Paediatric Surgery, Turku University Hospital, Kiinamyllynkatu 4-8 PL 52, FI-20521 Turku, Finland

**Keywords:** Cryptorchidism, PCBs, PCDD/Fs, Dioxins, PBDEs, Testis

## Abstract

**Background:**

Congenital cryptorchidism, i.e. failure of the testicular descent to the bottom of the scrotum, is a common birth defect. The evidence from epidemiological, wildlife, and animal studies suggests that exposure to mixtures of endocrine disrupting chemicals during fetal development may play a role in its pathogenesis. We aimed to assess the association between cryptorchidism and prenatal exposure to polychlorinated biphenyls (PCBs), polychlorinated dibenzo-*p*-dioxins and furans (PCDD/Fs), and polybrominated diphenyl ethers (PBDEs).

**Methods:**

We conducted a case–control study consisting of 44 cryptorchid cases, and 38 controls operated for inguinal hernia, umbilical hernia, or hydrocele at the Turku University Hospital or Rigshospitalet, Copenhagen in 2002–2006. During the operation a subcutaneous adipose tissue biopsy was taken. Samples were analysed for 37 PCBs, 17 PCDD/Fs and 14 PBDEs by gas chromatography-high-resolution mass spectrometry. Chemical concentrations were adjusted for postnatal variation introduced by differences in duration of breastfeeding, age at the operation, and country of origin with a multiple linear regression. Association between adjusted and unadjusted chemical concentrations and the risk of cryptorchidism were analysed with logistic regression to get an estimate for odds ratio (OR) of cryptorchidism per multiplication of chemical concentrations with ca. 2.71 (Napier’s constant).

**Results:**

Total-TEq i.e. the WHO-recommended 2,3,7,8-TCDD equivalent quantity of 17 dioxins and 12 dioxin-like PCBs and sum of PCDD/Fs were positively associated with cryptorchidism [OR 3.21 (95 % CI 1.29–9.09), OR 3.69 (95 % CI 1.45–10.9), respectively], when adjusting for country of origin, the duration the child was breastfed, and age at operation. The association between the sum of PCBs and cryptorchidism was close to significant [OR 1.92 (95 % CI 0.98–4.01)], whereas the association between the sum of PBDEs and cryptorchidism was not [OR 0.86 (95 % CI 0.47–1.54)]. There were no associations between unadjusted chemical concentrations and the risk of cryptorchidism.

**Conclusions:**

Prenatal exposure to PCDD/Fs and PCDD/F-like PCBs may be associated with increased risk for cryptorchidism. Our finding does not exclude the possibility of an association between the exposure to PBDEs and cryptorchidism.

**Electronic supplementary material:**

The online version of this article (doi:10.1186/s12940-015-0065-0) contains supplementary material, which is available to authorized users.

## Introduction

According to prospective cohort studies, congenital cryptorchidism i.e. failure of the testicular descent to the bottom of the scrotum is found in 1.8–8.4 % of mature new-born boys [1]. It is a well-established risk factor for testicular cancer and is associated with compromised fertility [2, 3]. The incidence of congenital cryptorchidism has apparently increased in Denmark and the UK according to prospective cohort studies using similar diagnostic criteria [4, 5]. Based on epidemiological, experimental, and wildlife evidence, recently reviewed by the UNEP/WHO expert panel [6], it has been hypothesised that the prenatal exposure to anti-androgenic endocrine disrupting chemicals might be causally related to congenital cryptorchidism [7]. Further research in animal models suggests that the developing male fetus is especially susceptible to the anti-androgenic effects during 8–14 weeks of gestation when the development of the male reproductive tract appears to be programmed [8].

Persistent organic pollutants are a group of potential endocrine disrupting chemicals that are toxic, persistent, able to bioaccumulate, and move long distances by natural processes in soil, water, air, and biota [6]. This group includes intentionally produced chemicals such as polychlorinated biphenyls (PCBs) that were banned in the mid-1980s and polychlorinated dibenzo-*p*-dioxins and dibenzofurans (PCDD/Fs or “dioxins”), which are unintentionally produced during combustion and industrial processes [6, 9]. Unlike these two persistent organic pollutants, some polybrominated diphenyl ether (PBDE) congeners are still actively produced and used as flame-retardants in electrical devices, furniture, and textiles [6]. US citizens are substantially more exposed to PBDEs compared to Europeans, very likely due to differences in the fire safety standards [10].

In Northern Europe, exposure studies show declining body burdens of PCBs and PCDD/Fs after the 1980s whereas the body burden of PBDEs kept increasing rapidly until the early 2000s [11, 12]. Today, humans are exposed to PCBs, PCDD/Fs, and PBDEs via breastfeeding and consumption of fish, meat, and dairy products [13–15], and to PBDEs also via inhalation and ingestion of indoor dust [16]. PCBs, PCDD/Fs, and PBDEs are excreted to lipid-rich breast milk, and the exposure per kilogram body weight is high during lactation and in toddlers [14, 16].

In experimental studies with laboratory animals, there is evidence of adverse changes in the reproductive system of the offspring after maternal exposure to PCBs, PCDD/Fs and PBDEs [17–21], including some reports that maternal exposure to PCBs and PCDD/Fs, but not to PBDEs, may disrupt or delay testicular descent [22–25]. An occupational study suggested already in 1996 that the exposure of male sawmill workers to PCDD/F-contaminated chlorophenol was associated with congenital cryptorchidism in their male offspring [26]. In addition, previous epidemiological studies suggested a relatively weak association between cryptorchidism and levels of PCBs, PCDD/Fs, and PBDEs in breast milk [27–29]. However, other studies examining maternal serum, placenta, umbilical cord or cord blood, which have smaller lipid content, did not observe the same associations [28–32]. In two of our previous studies, we also observed a positive correlation between serum LH levels and the exposure to PCBs and PBDEs, suggesting a subtle effect on testicular Leydig cell function [28, 30].

As a lipid-rich matrix, adipose tissue provides a good alternative for examining these fat-soluble persistent organic pollutants in the child. The lipid-adjusted concentrations of persistent organic pollutants appear to be similar between serum and adipose tissue [33], but the serum levels appear to have a higher day-to-day variation due to dietary exposure to persistent organic pollutants [34]. Furthermore, due to high lipid content in adipose tissue, concentrations of these chemicals can be detected from adipose tissue at the level that would require high volumes of blood to be drawn from the small child. In contrast, if the child is operated for a medical condition, fat tissue biopsies can be taken during the operation without further risk or distress to the child. To our knowledge, only one study has examined the association between cryptorchidism and the levels of various persistent organic pollutants in the adipose tissue; Hosie et al. found that the concentrations of heptachloroepoxides and hexachlorobenzenes, but not PCBs, were higher in cryptorchid boys than in controls [35]. However, some of the subjects in that study were more than 10 years old, which increases the postnatal exposure, and therefore decreases the likelihood of observing a difference between groups.

To assess the association between congenital cryptorchidism and exposure to PCBs, PCDD/Fs and PBDEs during pregnancy, we carried out a non-matched cross-sectional case–control study comparing the levels of PCBs, PCDD/Fs and PBDEs between boys operated for cryptorchidism and for inguinal hernia, abdominal hernia or hydrocele. Adipose tissue concentrations of PCBs, PCDD/Fs, and PBDEs were used as a proxy for cumulative prenatal chemical exposure.

## Methods

### Study design

The participants were recruited from the children operated in the Departments of Paediatric Surgery in Turku University Hospital, Turku, Finland (2002–2006) and Rigshospitalet, Copenhagen, Denmark (2004–2005). In Finland, children under 5 years of age were included. In Denmark, there were no age limitations, but parents and grandparents of the child had to be born and raised in Denmark and not have lived abroad for more than 3 years (mother) or 10 years (rest of the family). Although there were no limitations considering ethnicity in the Finnish study, all participants were of Finnish origin. Thirty-five Finnish and 14 Danish boys who were scheduled for orchidopexy of one or both testes by a paediatric surgeon were included as cases. Five of the Finnish cases were excluded from the study when no testis was found at operation. Twelve of the Finnish and two of the Danish cases were referred to orchidopexy from a prospective study to assess the prevalence of congenital cryptorchidism [5]. Twenty-seven Finnish boys who were operated for inguinal hernia, one for abdominal hernia, and one for hydrocele, and nine Danish boys who were operated for inguinal hernia were recruited as controls. During the operation, a subcutaneous adipose tissue biopsy was taken from the incision area and samples were frozen. The location of the biopsy was same both among cases and controls.

Main outcome measures were sums of 37 PCBs, 17 PCDD/Fs, 14 PBDEs, and total-TEq, i.e. WHO-recommended 2,3,7,8-TCDD equivalent quantity of 17 dioxins and 12 dioxin-like PCBs, in adipose tissue. The TEq value was calculated by multiplying the concentration of each congener with a toxic equivalency factor [36], and summing up all equivalents.

Before the operation, the parents of the Finnish subjects were asked to answer a questionnaire how many months the child was breastfed exclusively, the age when the breastfeeding was discontinued, and the total number of months the mother had breastfed her previous children. Obstetric data of 28 Finnish cases and 22 controls (maternal smoking, gestational diabetes, length of gestation, mother’s weight before pregnancy, birth weight, parity) were obtained from medical records, while data of two cases and seven controls were not available. If maternal smoking or gestational diabetes were not mentioned in the medical records, the mother was assumed to be a non-smoker and/or without gestational diabetes. Danish versions of the questionnaire that additionally contained questions on length of gestation, gestational diabetes, and birth weight were mailed to the parents of the subjects in 2013, and those who did not answer were contacted via telephone and interviewed. The Danish subjects were not asked about the occurrence of maternal smoking. Three Danish cases and one control answered to neither mailed questionnaire nor telephone call. Altogether 24 cases (nine Danish, 15 Finnish) and 28 (eight Danish, 20 Finnish) controls had all the background data available except for maternal smoking.

### Ethics statement

The study was performed according to the Helsinki II declaration and approved by the Joint Ethics Committee of the University of Turku and Turku University Hospital, the Local Danish Ethics committee and the Danish Data Protection Agency. Parents gave oral and written informed consent.

### Chemical analyses

Frozen samples were sent to the National Institute for Health and Welfare, Department of Environmental Health in Kuopio, Finland. Seventeen PCDD/F congeners, 37 PCB congeners, and 14 PBDE congeners (listed in Table 1) were selected for the analyses. In the laboratory, samples were defrosted and weighed. The weight of a single tissue biopsy ranged from 0.043 to 0.78 g. The procedures to extract, fractionate, and purify a sample and analyses by high-resolution mass-spectrometer have been described in detail previously [37–39]. Due to the small sample size it was not possible to determine the fat content, and therefore concentrations are given as nano- or picograms per gram sample. The case and control samples were analysed in a randomly mixed order in seven batches and blinded for the technician and chemist.

**Table 1:** Persistent organic pollutants measured in the study

Persistent organic pollutants	Congeners
Polychlorinated dibenzo-*p*-dioxins and furans (PCDD/Fs)	2,3,7,8-TCDF, 2,3,7,8-TCDD, 1,2,3,7,8-PeCDF, 2,3,4,7,8-PeCDF, 1,2,3,7,8-PeCDF, 1,2,3,4,7,8-HxCDF, 1,2,3,6,7,8-HxCDF, 2,3,4,6,7,8-HxCDF, 1,2,3,7,8,9-HxCDF, 1,2,3,4,7,8-HxCDD, 1,2,3,6,7,8-HxCDD, 1,2,3,7,8,9-HxCDD, 1,2,3,4,6,7,8-HpCDF, 1,2,3,4,7,8,9-HpCDF, 1,2,3,4,6,7,8-HpCDD, OCDF, and OCDD
Polychlorinated biphenyls (PCBs)	18, 28/31, 33, 47, 49, 51, 52, 60, 66, 74, 77^a^, 81^a^, 99, 101, 105^a^, 110, 114^a^, 118^a^, 122, 123^a^, 126^a^, 128, 138, 141, 153, 156^a^, 157^a^, 167^a^, 169^a^, 170, 180, 183, 187, 189^a^, 194, 206, and 209
Polybrominated diphenyl ethers (PBDEs)	28, 47, 66, 71, 75, 77, 85, 99, 100, 119, 138, 153, 154, and 183

^a^PCDD/F-like PCB congener

Recoveries of individual internal PCDD/F, PCB, and PBDE standards were >60 %. Median limit of quantification (LOQ) of a single congener was 1.3 pg/g (range: 0.06–14 pg/g) for tetra- to heptachlorinated and 8.8 pg/g (range: 1.1–36 pg/g) for octachlorinated PCDD/F congeners. For non-*ortho*-PCBs median LOQ was 1.0 pg/g (range: 0.11–5.3 pg/g), and the median LOQ for mono- and di-*ortho*-PCBs was 0.05 ng/g (range: 0.003–3.9 ng/g). For PBDEs median LOQ was 0.048 ng/g (range: 0.003–2.9 ng/g). In the in-house fat control sample, the relative standard deviations for the sum of PCDD/Fs, the sum of PCBs, total-TEq, and the sum of PBDEs were 3.3, 7.2, 13, and 8.9 %, respectively.

Procedural blank samples were analysed to monitor laboratory and cross-sample contamination. The Finnish Accreditation Service, FINAS, has certified the laboratory (T077) in performing PCDD/F, PCB, and PBDE analyses in biological samples according to the EN ISO/IEC 17025 standard.

### Statistics

The data was analysed with the R statistical package, version 3.1.3 [40]. Values from the chemical analyses below LOQ were treated as LOQ/2 and missing data as system missing. Group differences in the incidence of gestational diabetes, parity, and maternal smoking were tested with Fischer’s exact test. Differences between groups in normally distributed demographic variables were tested with independent samples *T*-test (maternal age, total duration of breastfeeding, duration of exclusive breastfeeding), with independent samples *T*-test after logarithmic transformation (mothers’ weight before pregnancy, age at the operation), or with Mann–Whitney *U*-test (previous breastfeeding, length of gestation, birth weight).

Total-TEq and sums of PCBs, PCDD/Fs, and PBDEs were calculated. Only TEq and sums were analysed to avoid mass significance of multiple comparisons. Furthermore, the concentrations of most chemicals within one group correlated with each other (data not shown), and it is therefore not possible to distinguish their individual effects. Correlations between predictors and sum of PCBs, sum of PCDD/Fs, and sum of PBDEs were tested with Spearman’s rank correlation coefficient. Unadjusted differences in chemical levels and TEqs between cases and controls in Finland and Denmark were estimated with independent samples *T*-test after logarithmic transformation. Association between ln transformed levels of PCBs, PCDD/Fs, and PBDEs and the risk of congenital cryptorchidism was assessed with a logistic regression analysis, which also provided the odds ratios (ORs) i.e. the increase of odds of cryptorchidism per multiplication of chemical concentration with ca. 2.71 (Napier’s constant).

A two-stage testing strategy was chosen, in which first the chemical concentrations were first adjusted for postnatal exposure by linear regression. Then the associations between the adjusted chemical concentrations and the risk of congenital cryptorchidism were tested with an unadjusted logistic regression, providing the ORs as in unadjusted analyses, but for chemical concentration which was not explained by duration of breastfeeding, age at the operation, and country of origin.

**Fig. 1:**
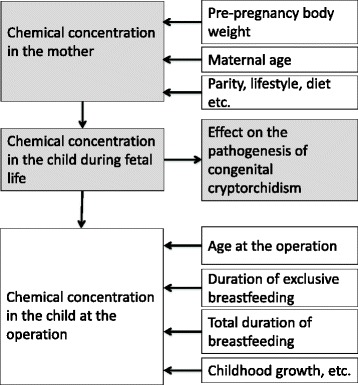
Factors contributing to the prenatal and postnatal concentration of chemicals and their relationship with the pathogenesis of congenital cryptorchidism. Chemical concentrations in the analyses were adjusted for total duration of breastfeeding and age at the operation to minimise the variation in the postnatal exposure, which does not contribute to the pathogenesis of congenital cryptorchidism. In contrast, factors influencing the chemical concentrations in the mothers were not adjusted for to maximise the likelihood to detect the association between the chemical concentrations and the outcome

The selection of the adjustment variables in the first stage is shown in Fig. 1. According to a previous line of research, the male reproductive development is programmed during fetal life [8]. Therefore, we hypothesise that the exposure before birth was causative, and the postnatal exposure was not. Consequently, we aimed to minimise the variation in the postnatal exposure by adjusting for the exposure via breastfeeding [13–16], whereas the predictors of the prenatal exposure were left unadjusted. Thus, ln transformed chemical concentrations were adjusted for the age when breastfeeding was discontinued, age at the operation, and the country of origin in a linear regression model, but not adjusted for maternal age or parity. Regression coefficients were transformed back from ln values to calculate a) the relative increase (percentage) in chemical concentration for each month of breastfeeding or higher age at operation and b) the mean ratio of chemical concentrations in the two countries (Denmark/Finland).

Previous studies suggest that gestational diabetes and preterm birth may independently increase the risk of cryptorchidism [41–43], and may be related to the level of the exposure at or before birth. Thus, the analyses were repeated excluding those children (11 cases, 12 controls), who were born before gestational week 37 + 0, whose mothers had gestational diabetes, or who were older than 5 years old. *P*-values below 0.05 were considered statistically significant.

## Results

### Participants

Study sample characteristics are presented in Table 2. Forty cases (11 Danish, 29 Finnish) and 33 controls (eight Danish, 25 Finnish) had data from chemical measurements, duration of breastfeeding and age at the operation available for the analyses. The mothers of the cryptorchid boys were older than those of controls (*p* = 0.04), and had a longer gestation (*p* = 0.04). There were no significant group differences in gestational diabetes, parity, maternal smoking, total number of months the mother had breastfed in previous pregnancies, weight before pregnancy, birth weight, the duration of exclusive breastfeeding, age when the breastfeeding was discontinued, or age at operation. Four and two boys in case and control group, respectively, were not breastfed at all.

**Table 2:** Study sample characteristics

	Cases N (%) or mean ± SD, IQR	Controls N (%) or mean ± SD, IQR	Missing case, control	p^a^
Total N	44	38		
Gestational diabetes	8 (21 %)	4 (13 %)	5, 7	0.58
Parity			3, 1	0.24
0	23 (56 %)	15 (41 %)		
1	7 (17 %)	13 (35 %)		
2	9 (22 %)	6 (16 %)		
3	2 (5 %)	3 (8 %)		
Maternal smoking during pregnancy	5 (18 %)	2 (9 %)	16, 16	0.47
Maternal age (years)^*^	32.3 ± 4.9,	29.6 ± 6.3,	1, 0	0.04^*^
	28.7–35.1	24.5–35.1		
Months breastfed in previous pregnancies	3.4 ± 5.8,	6.2 ± 8.8,	14, 8	0.51
	0–4.0	0–10.8		
Mother’s weight before pregnancy (kg)	68.6 ± 16.7,	68.6 ± 19.7,	5, 8	0.92
	56.0–75.7	57.2–71.0		
Length of gestation (weeks)^*^	39.4 ± 2.9,	38.2 ± 3.0,	6, 7	0.04^*^
	38.8–41.0	37.6–40.6		
Birth weight (g)	3460 ± 680,	3170 ± 770,	5, 7	0.07
	3200–3875	2955–3630		
Duration of exclusive breastfeeding (months)	2.7 ± 2.1,	2.8 ± 2.1,	5, 4	0.87
	0.85–4.0	1.0–4.0		
Total duration of breastfeeding (months)	5.8 ± 4.5,	7.1 ± 5.7,	4, 5	0.26
	2.0–8.0	3.0–12.0		
Age at operation (years)	2.3 ± 1.0,	2.9 ± 2.2,	0, 0	0.53
	1.9–2.5	1.2–4.0		

*IQR* Interquartile range. ^a^For the difference between cases and controls. ^*^Statistically significant (*p* < 0.05)

### Levels of PCBs, PCDD/Fs, and PBDEs in adipose tissue

Concentrations of the total-TEq, and sums of PCBs, PCDD/Fs, and PBDEs are shown in Table 3. Number and percentage of individual PCB, PCDD/F, and PBDE congeners above the LOQ are given in Additional file 1. There were no differences between cases and controls in the unadjusted chemical concentrations in either of the countries. The sums of PCDD/Fs, PCBs, and total-TEq in adipose tissue correlated positively with the total duration of breastfeeding (r_s_ = 0.58, *p* < 0.001; r_s_ = 0.79 *p* < 0.001; and r_s_ = 0.78, *p* < 0.001, respectively), duration of exclusive breastfeeding (r_s_ = 0.51, *p* < 0.001; r_s_ = 0.71, *p* < 0.001; and r_s_ = 0.68, *p* < 0.001, respectively), and maternal age (r_s_ = 0.28, *p* = 0.002; r_s_ = 0.34, *p* = 0.01; and r_s_ = 0.30, *p* = 0.007, respectively). The sum of PCBs and total-TEq also correlated with the length of gestation (r_s_ = 0.28, *p* = 0.02 and r_s_ = 0.31, *p* = 0.009, respectively), and the sum of PCBs with age at operation (r_s_ = 0.23, *p* = 0.03). The sum of PBDEs correlated positively only with parity and duration of previous breastfeeding (r_s_ = 0.36, *p* = 0.03 and r_s_ = 0.40 *p* = 0.03, respectively). Birth weight and mothers’ weight before pregnancy were not associated with levels of PCBs, PCDD/Fs, PBDEs, or total-TEq in adipose tissue.

**Table 3:** Sums of PCBs, PCDD/Fs and PBDEs, and total-TEq in cryptorchid and healthy boys

	Cases median (range)	Controls median (range)	p^a^
Finland	*N* = 30	*N* = 29	
Sum of PCDD/Fs (pg/g)	113 (41.5–320)	77.7 (27.9–1290)	0.25
Sum of PCBs (ng/g)	68.9 (12.7–389)	80.0 (11.2–570)	0.97
Sum of PBDEs (ng/g)	7.01 (1.56–63.6)	5.41 (1.28–85.9)	0.42
Total-TEq (pg/g)	7.44 (3.24–40.7)	5.43 (2.65–64.1)	0.85
Denmark	*N* = 14	*N* = 9	
Sum of PCDD/Fs (pg/g)	88.9 (21.7–258)	83.0 (21.7–164)	0.83
Sum of PCBs (ng/g)	182 (17.4–698)	164 (12.8–517)	0.56
Sum of PBDEs (ng/g)	3.94 (1.56–31.0)	5.67 (3.54–9.43)	0.45
Total-TEq (pg/g)	18.5 (3.41–56.0)	13.0 (2.64–42.0)	0.73
Finland and Denmark	*N* = 44	*N* = 38	
Sum of PCDD/Fs (pg/g)	103 (21.7–320)	79.1 (21.7–1290)	0.94
Sum of PCBs (ng/g)	97.0 (12.7–698)	134 (11.2–570)	0.73
Sum of PBDEs (ng/g)	4.90 (1.56–63.6)	5.54 (1.28–85.9)	0.26
Total-TEq (pg/g)	9.78 (3.24–56.0)	7.50 (2.64–64.1)	0.55

^a^For the difference between cases and controls

The results of the linear regression analysis are presented in Table 4. For each month of breastfeeding the levels of total-TEq, sum of PCDD/Fs and sum of PCBs increased with 13, 8, and 16 %. Duration of breastfeeding explained 48, 27, and 44 %, respectively, of the variation in the concentrations of total-TEq, sum of PCDD/Fs and sum of PCB. Duration of breastfeeding did not correlate with the sum of PBDEs in our model. When adjusting for breastfeeding and age at the operation, Danish boys had 39 % smaller sum of PCDD/Fs and 47 % smaller sum of PBDEs than Finnish, whereas there were no significant differences in the total-TEq or sum of PCBs. Age at the operation did not correlate with any of the chemical concentrations, when adjusting for duration of breastfeeding and country of origin.

**Table 4:** Adjustments for postnatal exposure to PCBs, PCDD/Fs and PBDEs using a multiple linear regression

		Sum of PCDD/Fs	Sum of PCBs	Sum of PBDEs	Total-TEq
Country^a^	β (95 % CI)^b^	0.61 (0.43–0.87)	1.20 (0.79–1.81)	0.53 (0.33–0.83)	1.13 (0.81–1.57)
	p	0.006	0.38	0.007	0.46
	R^2^	0.08	<0.01	0.10	<0.01
Duration of breastfeeding^a^	β (95 % CI)^c^	1.08 (1.05–1.12)	1.16 (1.12–1.21)	1.01 (0.97–1.05)	1.13 (1.10–1.17)
	p	<0.001	<0.001	0.70	<0.001
	R^2^	0.27	0.44	0.05	0.48
Age at the operation^a^	β (95 % CI)^d^	1.00 (0.99–1.01)	1.00 (0.99–1.01)	1.01 (1.00–1.02)	1.00 (0.99–1.00)
	p	0.73	0.83	0.27	0.27
	R^2^	<0.01	<0.01	0.02	<0.01

^a^Altogether 40 cases and 33 controls. ^b^Regression coefficients for the ratio of mean chemical concentrations in Danish versus Finnish subjects, adjusted for duration of breastfeeding and age at the operation. ^c^Regression coefficients for the relative increase in adjusted chemical concentrations per month of breastfeeding, adjusted for country of origin and age at operation. ^d^Regression coefficients for the relative increase in chemical concentrations per month of age at operation, adjusted for country of origin and duration of breastfeeding

### Exposure to PCBs, PCDD/Fs and PBDEs and the risk of cryptorchidism

The ORs with 95 % confidence intervals (95 % CI) for the association between concentrations of PCBs, PCDD/Fs and PBDEs and the risk of cryptorchidism are presented in the Table 5. In the unadjusted logistic regression, there were no significant associations. However, when adjusting for the country of origin, age at operation and the duration of breastfeeding, total-TEq (OR 3.21, 95 % CI 1.29–9.09, *p* = 0.02), and sum of PCDD/Fs (OR 3.69, 95 % CI 1.45–10.9, *p* = 0.01) were associated with and an increased risk of cryptorchidism, while the association of cryptorchidism with the sum of PCBs was close to significant (OR 1.92, 95 % CI 0.98–4.01, *p* = 0.07). The sum of PBDEs was not associated with the risk of cryptorchidism (OR 0.86, 0.47—1.54, *p* = 0.61).

**Table 5:** ORs for the association between congenital cryptorchidism and adjusted^c^ sums of PCDD/Fs, PCBs, PBDEs, and Total-TEq

	Unadjusted^a^		Adjusted^b,c^	
	Odds ratio (95 % CI)	p^d^	Odds ratio (95 % CI)	p^d^
Sum of PCDD/Fs	1.41 (0.79–2.61)	0.25	3.69 (1.45–10.9)	0.01
Sum of PCBs	1.04 (0.70–1.54)	0.84	1.92 (0.98–4.01)	0.07
Sum of PBDEs	0.91 (0.53–1.55)	0.73	0.86 (0.47–1.54)	0.61
Total-TEq	1.17 (0.71–1.93)	0.54	3.21 (1.29–9.09)	0.02

^a^Altogether 44 cases and 38 controls. ^b^Altogether 40 cases and 33 controls. ^c^Adjusted for the country of origin, age at operation, and age when breastfeeding was discontinued. ^d^For the difference between cases and controls

Estimates did not change substantially when the analyses were restricted to boys who were born term, operated at less than 5 years of age, and whose mothers did not have gestational diabetes. In this sensitivity analysis, levels of total-TEq (OR 3.47, 95 % CI 1.15–12.9, *p* = 0.04) and sum of PCDD/Fs (OR 10.2, 95 % CI 2.36–65.5, *p* = 0.005) were positively associated with the risk of cryptorchidism, whereas sum of PCBs (OR 2.09, 95 % CI 0.82–6.04, *p* = 0.14) and sum of PBDEs (OR 0.80, 95 % CI 0.28–2.14, *p* = 0.64) were not.

## Discussion

Our study showed that exposure to PCDD/Fs and dioxin-like PCBs in adipose tissue increased the risk of cryptorchidism. However, this finding became statistically significant only when the country of origin, duration of breastfeeding and age at operation were included in the statistical analyses controlling for postnatal exposure. Furthermore, similar associations were noted when the analyses were restricted to children who were less than 5 years old at operation, born term, and whose mothers did not have gestational diabetes.

The concentrations of PCBs and PCDD/Fs in adipose tissue reflect the total cumulative exposure to these persistent substances both before and after birth. Adipose tissue biopsies were taken at the mean age of 2.3 and 2.9 years in the case and control groups, respectively, and there was no significant increase in the risk of cryptorchidism with increasing absolute levels of the chemicals in that age. However, as congenital cryptorchidism must arise from prenatal exposure, we further refined our analyses by controlling for variation in postnatal exposure. During the first several months the children are predominantly exposed to PCBs and PCDD/Fs via breast milk and later during childhood via ingestion of fatty foods [13, 14], and accordingly duration of breastfeeding could explain 27–48 % of the variation in the sums of PCBs and PCDD/Fs and total-TEq in our study. Therefore, the concentrations were adjusted for the difference of 1.3 months between cases and controls in duration of breastfeeding, which resulted in a significant difference in the risk of cryptorchidism. The analyses were also adjusted for country of origin and age at operation, but this did not have a substantial influence on the results compared to adjustment for duration of breastfeeding.

A child is predominantly exposed to PBDEs via breastfeeding and diet, but in highly exposed individuals, the exposure via dust and indoor air can represent up to 99 % of the total exposure to some PBDE congeners [16]. This substantial variation in the exposure via dust may explain why the sum of PBDEs did not correlate with most of the expected predictors in our study. Therefore, our linear regression analysis may have lacked the statistical power to find a correlation between the sum of PBDEs and age at operation, or total duration of the breastfeeding. Consequently, as highlighted by the small R^2^s of the linear regression model, our model was not able to adjust for postnatal exposure to PBDEs and our finding does not exclude the possibility of an association between cryptorchidism and exposure to PBDEs.

A recent study reported that persistent organic pollutant concentrations measured at the age of 6–11 years correlated poorly with maternal exposure levels, highlighting that the exposure later during childhood may compromise the assessment of the prenatal exposure [44]. However, the concentration of some congeners did correlate between mother and child even at that age, although the correlation was weak [44]. In addition to the aforementioned adjustments for duration of breastfeeding, age at operation, and country of origin, we took this into account in the supplemental analysis by excluding the boys who were older than 5 years when the fat biopsy was taken, which provided similar results.

Mortamais and co-workers have previously used a similar statistical approach, in which the authors first conducted a linear regression analysis adjusting for various factors in sampling and based on the results calculated the standardised chemical concentration under reference sampling conditions [45]. In this study we similarly adjusted for the variation with a linear regression and then continued by relating this adjusted concentration to the risk of cryptorchidism using logistic regression. However, in this study we were primarily interested in the relationship between exposure to PCBs, PCDD/Fs and PBDEs and congenital cryptorchidism instead of actual chemical levels and therefore we merely used the residuals of the first stage of analyses instead of the calculated estimate. The results of both studies suggest that the adjustment for the variation introduced by sampling conditions and the between-subject factors is necessary especially with small sample sizes, and failure to do so may result in significant associations being lost.

The mothers of the cryptorchid children were significantly older than those of the control group, despite the fact that they were both recruited randomly. This is in agreement with previous studies that have identified maternal age as a risk factor of congenital cryptorchidism [42, 43]. Interestingly, maternal age also correlated with increased levels of PCBs and PCDD/Fs in their offspring, which may contribute to the risk of cryptorchidism. We argue that when studying the association between congenital cryptorchidism and exposure to endocrine disrupting chemicals, this kind of predictors of prenatal exposure should not be adjusted for. Theoretically, if all the predictors of prenatal exposure to endocrine disrupting chemicals between the mothers were known and adjusted for, we would not find an association between cryptorchidism and exposure to endocrine disrupting chemicals even if there was a true relationship between the two. However, if maternal age has an independent effect on the pathogenesis of congenital cryptorchidism, which is not a consequence of the age-related increase in the body burden of the chemicals, it might explain some of the observed association between cryptorchidism and exposure to PCDD/Fs and PCDD/F-like PCBs.

To our knowledge, there is only one previous study examining the association between cryptorchidism and PCBs, PCDD/Fs, or PBDEs in adipose tissue biopsies. That study did not have information on breastfeeding of the boys and, similarly to our results, no association was found between the sum of six PCB congeners (all non-PCDD/F-like) and cryptorchidism in 18 cryptorchid children and 30 controls [35].

There is evidence that increased occupational exposure to dioxin-contaminated chlorophenol in a cohort of 9512 male saw mill workers between 1952 and 1988 was associated with an increased risk of cryptorchidism in their offspring [26]. Furthermore, in previous studies with other biological matrices the mothers of cryptorchid children in Denmark, but not in Finland, had higher sum of 14 PBDEs and WHO-TEq of 17 PCDD/Fs (no PCBs included in WHO-TEq) in breast milk [27, 28]. However, when the placental samples from Danish and Finnish mothers were analysed, there were no differences between cryptorchid and healthy children in the same groups of chemicals [28, 30].

The evidence for an association between cryptorchidism and exposure to PCBs appears weak. In a large US study comparing serum samples of mothers of 230 boys with cryptorchidism and 593 healthy children no association between cryptorchidism and sum of 11 PCBs (two PCDD/F-like, nine non-PCDD/F-like) could be demonstrated when controlling for serum concentration of dichlorodiphenyldichloroethylene (DDE) [32]. In addition, there were no differences in the sum of 37 PCBs (12 PCDD/F-like, 25 non-PCDD/F-like) in the aforementioned Danish-Finnish study in which placentas from mothers of 56 Finnish and 39 Danish cryptorchid and 56 Finnish and 129 Danish healthy children were analysed [30]. Furthermore, no difference was found in the sum of three PCBs (all non-PCDD/F-like) in umbilical cord samples between 20 cases (19 cryptorchid children, one testicular torsion) and 176 controls in the Faroe Islands [31].

Similarly, a French study found no statistically significant differences between cases and controls in the median sum of seven PCBs (one PCDD/F-like, six non-PCDD/F like) in cord blood (67 cryptorchid and 84 healthy children) or in breast milk from their mothers (mothers of 56 cryptorchid and 69 healthy children) [29]. However, compared to controls it was more common for cryptorchid children to have maternal breast milk levels above the median of quantifiable values [29]. In the Danish-Finnish study examining 37 PCBs in breast milk, levels of several non-PCDD/F-like congeners in breast milk of Danish subjects (29 cryptorchid cases, 36 controls) were associated with a decreased risk of congenital cryptorchidism, whereas in the Finnish subjects (33 cases, 32 controls) no such effect could be demonstrated [27].

Taken together, these results indicate that the effect of PCB exposure on the risk of cryptorchidism is too small to be distinguished from the effects of the total background exposure to other endocrine disrupting chemicals. However, this study and our previous results indicate that exposure to PCDD/Fs accompanied by PCDD/F-like PCBs that share the same toxicological pathway may contribute to testicular maldescent.

There are some weaknesses in our study. Firstly, our study is small and our control group included boys with inguinal hernia, which has been associated with cryptorchidism [42, 43]. However, since there are no data to suggest that boys with inguinal hernia would be less exposed than healthy children, it seems reasonable to assume that this shortcoming decreases rather than increases the chance of observing a group difference. Furthermore, since both cases and controls were recruited at departments of paediatric surgery, it is unsure if the association between cryptorchidism and chemical levels can be generalised to boys who have mild forms of cryptorchidism, which does not require operation. However, it is practically impossible to get inguinal adipose tissue biopsies from boys who would not be operated for a urogenital outcome on an ethically sound basis. Secondly, as the children were recruited to this study at various ages, our case group may include children with acquired undescended testes, and our control group may include boys with high scrotal testes or congenital cryptorchidism with spontaneous postnatal descent. Thirdly, we were not able to adjust for the lipid content of the samples, which increases the measurement error of the chemical concentrations. However, since the samples were taken directly from the adipose tissue, the variation in the lipid content should be small. Fourthly, the Danish mothers were asked about the duration of breastfeeding and obstetric data almost 10 years after the operation, which could raise questions about the accuracy of the data. However, there is no reason to believe that case or control group would remember the timing differently. Finally, we did not gather data on body size of the children, which may influence chemical concentrations in tissue samples, and thus increase the variance further. These limitations may have reduced group differences between cryptorchid cases and controls.

Strengths of our study include the high number of the PCB, PCDD/F and PBDE congeners analysed in the same subjects. Furthermore, the boys who had cryptorchidism were ‘true’ cases of cryptorchidism, since they were diagnosed both by the referring general practitioner and the paediatric surgeon, and did not descend spontaneously before orchidopexy. In addition, our study included boys from two countries, further increasing the generalizability of the results.

## Conclusions

Our study suggests that prenatal exposure to PCDD/Fs and PCDD/F-like PCBs is associated with cryptorchidism in humans. As there are geographical differences in the exposure pattern to endocrine disrupting chemicals, such association between cryptorchidism and the levels of PCBs and PCDD/Fs in adipose tissue may vary between different populations. The study cannot establish any causal relationship, but it supports the hypothesis that exposure to mixtures of endocrine disrupting chemicals may contribute to the aetiology of congenital cryptorchidism. Levels of PCBs and PCDD/Fs have declined substantially since the 1970s, which however is not paralleled by a declining incidence of cryptorchidism. This suggests that these chemicals are weak risk factors for cryptorchidism or other environmental risk factors have emerged during that window. Thus, PCBs and PCDD/Fs may represent a sentinel for other persistent organic pollutants with similar exposure sources.

## Additional file

Additional file 1: Table S1. Number and percentage of PCBs, PCDD/F, and PBDE congeners above LOQ (Limit of Quantification) in adipose tissue samples. (XLSX 43 kb)

## Abbreviations

PCBs: Polychlorinated biphenyls
PCDD/Fs: Polychlorinated dibenzo-*p*-dioxins and dibenzofurans
PBDEs: Polybrominated diphenyl ethers
LOQ: Limit of quantification
DDE: Dichlorodiphenyldichloroethylene
OR: Odds ratio
95 % CI: 95 % confidence interval
